# Educational pathways of childhood cancer survivors—a parental cohort

**DOI:** 10.1007/s11764-022-01303-y

**Published:** 2022-12-02

**Authors:** S. Michael, A. Borgmann-Staudt, G. Sommerhäuser, K. Kepakova, S. Klco-Brosius, J. Kruseova, E. Nagele, A. Panasiuk, J. Vetsch, M. Balcerek

**Affiliations:** 1 Department of Pediatric Oncology and Hematology, Corporate Member of Freie Universität Berlin, Humboldt-Universität Zu Berlin, and Berlin Institute of Health, Charité-Universitätsmedizin Berlin Germany, Augustenburger Platz 1, 13353 Berlin, Germany; 2 Department of Hematology, Oncology, and Cancer Immunology, Corporate Member of Freie Universität Berlin, Humboldt-Universität Zu Berlin, and Berlin Institute of Health, Charité-Universitätsmedizin Berlin Germany, Berlin, Germany; 3 University Hospital Brno, Brno, Czech Republic; 4 University Hospital Motol, Prague, Czech Republic; 5 Medical University Graz, Graz, Austria; 6 Medical University Wroclaw, Wroclaw, Poland; 7 Department of Health Sciences and Health Policy, University of Lucerne, Lucerne, Switzerland; 8 Berlin Institute of Health (BIH), Berlin, Germany

**Keywords:** University degree, Vocational training, Childhood cancer survivor, Offspring, Education

## Abstract

**Purpose:**

Using the International Standard Classification of Education (ISCED), we examined the educational and vocational pathways of two comparable, parental cohorts: childhood cancer survivors (CCS) and their siblings. Both cohorts had previously entered parenthood. The aim of the study was to elucidate whether childhood cancer and treatment affect the educational pathways chosen by parents who are former patients.

**Methods:**

We analysed data that was collected from childhood cancer survivors and their siblings regarding their offspring’s health within the *FeCt Multicentre Offspring Study* (conducted 2013–2016). We evaluated and compared the professional pathways of (i) all participating survivors and all participating siblings and those of (ii) survivors and their biological siblings.

**Results:**

Overall information on parental gender, age, and education were available from 1077 survivors and 246 siblings (group (i)). The majority of participants were female with a mean age of 35.2 (survivor) and 37.9 (sibling) years at time of survey. For subgroup (ii), analysis information was available on 191 survivors and 210 siblings. Fathers achieved university degrees significantly more often than mothers (*p* = 0.003 (i), *p* < 0.001 (ii)). The distribution of professional education was not significantly different between cancer survivors and siblings in either cohort (i) or (ii).

**Conclusions:**

Regarding our research on the educational and vocational trajectory of CCS, patients can be reassured that family planning and vocational education are well compatible. Inequalities regarding gender-specific educational pathways remain to be addressed.

**Implications for Cancer Survivors:**

CCS should monitor their fertility status regularly and, if necessary, cryopreserve germ cells or tissue in order to optimize their family planning. Educational opportunities should be pursued as desired and with confidence. Local as well as European aftercare programs can assist with family planning and education.

## Introduction

Advances in treatment have led to a consistent increase of survival rates in childhood and adolescent cancer patients [6]. This results in an increased proportion of patients who have survived for more than 5 years (survivors) within the general population [6]. However, a cure is not always synonymous with health. Treatment-related sequelae may occur during or immediately after treatment, but can also develop decades later. Infertility is a common treatment-related sequela [2, 16]. All treatment-related sequelae require individual, lifelong follow-up care for survivors. The St. Jude Lifetime Cohort Study reported a high percentage of late-effects (> 95%) among adult survivors of childhood cancer at age 45 [11]. However, overall, survivors appear to adjust well in adulthood and report health-related quality of life comparable to their peers [28]. This includes reports of positive mental health and life satisfaction [38], positive changes in self-esteem, relationships, and post-cancer life plans [3]—possibly indicating post-traumatic growth [3, 27].

The potential impact of cancer and its treatment on cognitive functioning and academic achievement have also become increasingly important [9]. The attainment of higher education in the form of degrees or certificates is closely linked to life chances. A European comparison of educational outcomes revealed country-specific differences; it reported that in contemporary Europe, women obtain a university degree more often than men [32]. Studies that have examined the educational and occupational performance of long-term survivors of pediatric cancer show heterogeneous results due to varying contexts. In a nationwide survey, former cancer patients were significantly more likely to complete A-level qualifications than the general German population. Participants in this survey were more often female [24]. Cancer type and treatment also have a decisive influence on educational pathways. For example, leukemia patients whose treatment included cranial irradiation had lower overall academic performances [9, 15]. Survivors at risk of poorer educational outcomes in recent studies also included those treated with cranial irradiation and those diagnosed with brain tumors or epilepsy [21, 35]. A US study showed that former cancer patients attained lower educational levels than their siblings. However, the sibling collective was significantly older at time of study conduction [7]. Cancer diagnosis and treatment also impact the developmental environment of healthy siblings: While children with cancer usually receive special attention and support from their parents, siblings often receive less—resulting in an increased risk for stress [17].

Family planning plays an equally large part in life planning. A recent study showed that patients with an increased risk of infertility more often achieved a university degree [16]. This connection between educational pathways and family planning was also identified in a state-wide survey conducted in Germany: Among individuals with a higher level of education, the proportion of those having children was lower compared to people with rudimentary or intermediate education. Young adults saw a risk in having children at a young age and feared that it would be more difficult to complete vocational training while raising children [5]. For childhood cancer survivors, special attention needs to be given within this context, as often a reduced fertile window exists which may close if educational achievements are chronologically prioritized.

### *Objective*

We examined degrees of professional training attained by former pediatric cancer patients who had previously entered parenthood and compared those achieved by their healthy siblings who likewise had biological children.

## Methods

### Study design and setting

We analyzed data regarding the educational pathways of pediatric cancer survivors and their siblings who participated in the *FeCt Multicentre Offspring Study*, which was conducted as an explorative, retrospective cohort study in Austria, Czech Republic, Germany, Poland, and Switzerland from 2013 to 2016. Health aspects in offspring born to childhood cancer survivors compared to those born to survivor siblings were surveyed using a questionnaire based on the Robert Koch Institute’s Child Health Survey Questionnaire [25]. It included a total of 46 items on diseases, well-being, living conditions, health behavior, and use of medical services, as well as socio-demographic information [4]. Study design and methods, including participant characteristics and aspects of offspring health, were previously published [31]. The study was approved by the local ethics committees of participating centers (lead votes Charité Universitätsmedizin Berlin, EA2/237/05 and EA2/103/11).

### Variables

For our analyses, information from survivors and siblings on gender, academic/vocational training, country of origin/migration background, employment status, and age at study entry was selected. Academic/vocational training was mapped using the internationally comparable ISCED classification [36] and categorized as: *no education*, *current education*, *non-university education* (ISCED 4–5) and *university education* (ISCED 6–8). From a total of 46 questionnaire items, information on gender, vocational training, country of origin, employment status, migration background, and age at study entry was selected and/or calculated to answer the study questions. The definition of a migration background was fulfilled if participants themselves, their parents, or their grandparents were born outside the country of study conduction. Cancer diagnosis (leukemia/lymphoma, solid tumors, brain tumors); treatment (chemotherapy, radiotherapy); and age at diagnosis were obtained from national registries and medical records.

### Participants and collective

The *FeCt Multicentre Offspring Study* comprises 1126 survivors and 271 siblings. As previously described, participating survivors from the *FeCt Multicentre Offspring Study* were more often female (*p* < 0.007), significantly older at the time of survey (*p* < 0.001), diagnosed between 1980 and 1999 (*p* < 0.001), and had received chemotherapy (*p* < 0.001) more often than non-respondents. No sibling non-responder analysis could be performed; as in most cases, participation was requested by survivors [31].

To increase comparability between survivors and siblings, cases with missing values on parental gender, age, and education were excluded (case-by-case exclusion). This resulted in a total of 1077 survivors and 246 siblings—group (i). For subgroup analyses on survivors with biological siblings—group (ii), we selected 191 survivors with 210 biological siblings.

### Statistical methods

The dataset was divided into the two groups: (i) the overall collective of all cancer survivors and siblings, and (ii) cancer survivors and biological siblings.

Analyses were carried out with IBM SPSS Statistics, version 27. In both groups, (i) and (ii), we descriptively examined how cancer survivor characteristics differed from those of the siblings (Table 1) and how these characteristics differed according to the attainment of a university degree (Table 2). Nominal and ordinal variables were presented as absolute and relative proportions. For the metric variable *age at time of survey*, the mean, median, standard deviation (SD), and interquartile range (IQR) were calculated. Significances were tested using the two-sided chi-square test for nominal variables, Pearson’s correlation for ordinal variables, and Spearman’s correlation for metric variables, with a significance level of < 5% to detect group differences.

We explored factors that potentially influence educational pathways in group (ii) using a generalized estimating equation (GEE) that modelled gender, age at time of survey, and survivor versus sibling. The generalized estimating equations extend the generalized linear model to account for analysis such as data grouped into clusters, which is the case in this study. As a subject variable, we created a variable that has the same value for survivors and siblings from the same family. For the covariance matrix, we chose the default robust estimator ([12], p. 1). As type of model, we chose the “Ordinal logistic,” since the dependent variable “vocational training” was considered ordinal (Table 3).

## Results

**Fig. 1:**
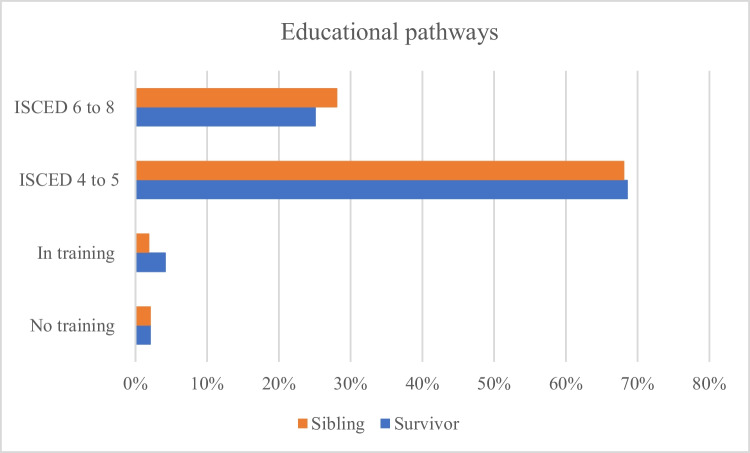
Educational pathways of childhood cancer survivors and their biological and non-biological siblings. ISCED 4–5, including vocational schools, polytechnic schools, programs at training institutions, master craftsman training; ISCED 6–8, including bachelor’s, master’s, doctoral, or equivalent level

Characteristics of survivors and their siblings with information on educational pathways are shown in Table 1 for the respective groups (i and ii) and Fig. 1 for group (ii). Both, country of origin (*p* < 0.001) and age at time of survey (*p* < 0.001), differed significantly between the groups (i), and survivors were younger than siblings at time of survey with an average of 35.2 versus 37.9 years. Overall, in both groups, (i) and (ii), siblings were less often not employed (retired, in training, etc.) and unemployed and were more often fully employed (*p* = 0.011 and *p* = 0.004).

**Table 1:** Characteristics of childhood cancer survivors and their biological and non-biological siblings

Characteristics	Total cohort (i)			Survivor and biological sibling (ii)		
	Survivors	Siblings	*p*	Survivors	Siblings	*p*
	*n* (%)	*n* (%)		*n* (%)	*n* (%)	
Total	1077 (100.0)	246 (100.0)		191 (100.0)	210 (100.0)	
Gender			0.315			0.104
Female	749 (69.5)	163 (66.3)		139 (72.8)	137 (65.2)	
Male	328 (30.5)	83 (33.7)		52 (27.2)	73 (34.8)	
Vocational training			0.336			0.334
No training (no completed degrees or certificates)	21 (1.9)	5 (2.0)		4 (2.1)	4 (1.9)	
In training (academic or vocational)	40 (3.7)	5 (2.0)		8 (4.2)	4 (1.9)	
Higher Academic Education ISCED 4 bis 5^a^	736 (68.3)	166 (67.5)		131 (68.6)	143 (68.1)	
ISCED 6 bis 8^b^	280 (26.0)	70 (28.5)		48 (25.1)	59 (28.1)	
Country of origin			** < 0.001**			0.338
Germany	836 (77.6)	162 (65.9)		136 (71.2)	143 (68.1)	
Austria	58 (5.4)	21 (8.4)		11 (5.8)	13 (6.2)	
Poland	15 (1.4)	1 (0.4)		-	-	
Switzerland	47 (4.4)	29 (11.8)		14 (7.3)	22 (10.5)	
Czech Republic	121 (11.2)	33 (13.4)		30 (15,7)	32 (15.2)	
Employment status			**0.011**			**0.004**
Not employed (retired, in training, etc.)	72 (6.7)	10 (4.1)		13 (6.8)	7 (3.4)	
Unemployed	35 (3.3)	3 (1.2)		6 (3.1)	-	
Temporary leave of absence (i.e., maternity leave))	155 (14.5)	32 (13.2)		31 (16.2)	28 (13.5)	
Part-time employment	358 (33.6)	79 (32.5)		64 (33.5)	64 (30.9)	
Full-time employment internship (i.e., apprenticeship)	442 (41.4)	118 (48.6)		75 (39.3)	107 (51.7)	
	5 (0.5)	1 (0.4)		2 (1.0)	1 (0.5)	
Age at time of survey			** < 0.001**			0.346
Mean age [SD]	35.22 [5.3]	37.85 [6.6]		37.03 [5.7]	37.61 [6.5]	
Median [IQR]	34.54 [9]	37.14 [11]		36.60 [9]	36.96 [11]	

(*p* < 0.05) are in bold

*SD* standard deviation, *IQR* interquartile range^a^Including vocational schools, polytechnic schools, programs at training institutions, master craftman’s training^b^Including bachelor’s, master’s, doctoral, or equivalent level

Distribution of characteristics among survivors and their siblings differed depending on the attainment of a university degree (Table 2). Male study participants achieved a university degree significantly more often than females in both groups (*p* = 0.003, group (i) and *p* < 0.001, group (ii)). Survivors and siblings achieved university degrees at similar rates. In group (ii), there was a significant correlation between country of origin and attainment of a university degree (*p* = 0.049). Participants with a migration background achieved a university degree significantly more often (group (i): *p* = 0.034; group (ii): *p* = 0.002). There were no significant differences between type of diagnoses, age at diagnosis, and year of diagnosis, with regard to attainment of a university degree.

**Table 2:** Descriptive analyses of university degree attainment by childhood cancer survivors and their siblings

Characteristics	Total cohort (i)			Survivors and biologic siblings (ii)			
University degree	no, *n* (%)	yes *n* (%)	*p*	no *n* (%)	yes *n* (%)		*p*
Total	973 (100.0)	350 (100.0)		294 (100.0)	107 (100.0)		
Gender			**0.003**				** < 0.001**
Female	693 (76.0)	219 (24.0)		216 (78.3)		60 (21.7)	
Male	280 (68.1)	131 (31.9)		78 (62.4)		47 (37.6)	
Survivor/sibling			0.431				0.503
Survivors	797 (74.0)	280 (26.0)		143 (74.9)		48 (25.1)	
Siblings	176 (71.5)	70 (28.5)		151 (71.9)		59 (28.1)	
Country of origin			0.276				**0.049**
Germany	723 (72.4)	275 (27.6)		196 (70.3)		83 (29.7)	
Austria	64 (81.0)	15 (19.0)		20 (83.3)		4 (16.7)	
Poland	5 (31.3)	11 (68.8)		-		-	
Switzerland	61 (80.3)	15 (19.7)		30 (83.3)		6 (16.7)	
Czech Republic	120 (77.9)	34 (22.1)		48 (77.4)		14 (22.6)	
Migrant background (at least one family member not born in respective country of survey)			**0.034**				**0.002**
Yes	191 (68.5)	88 (31.5)		55 (60.4)		36 (39.6)	
No	777 (74.8)	262 (25.2)		238 (77.0)		71 (23.0)	
Diagnoses			0.364				0.601
Leukemias/lymphomas	457 (74.6)	156 (25.4)		82 (71.3)		33 (28.7)	
Brain tumors	53 (74.6)	18 (25.4)		7 (63.6)		4 (36.4)	
Other solid tumors	255 (71.8)	100 (28.2)		46 (75.4)		15 (24.6)	

(*p* < 0.05) are in bold

The generalized estimating equation showed that of the variables studied (patient versus sibling, gender and age at inclusion), male gender was the main factor influencing academic/vocational training (OR 2.262 (1.433 to 3.571) *p* < 0.001, group (ii), Table 3).

**Table 3:** Generalized estimating equation for the confounders of academic/vocational training

Characteristics	aAcademic/vocational training	
	OR (95% CI)	p
Siblings	1.163 (0.801–1.689)	0.426
Survivors	Reference	
Male gender	2.262 (1.433–3.571)	**< 0.001**
Female gender	Reference	
Age at time of survey	1.000 (0.962–1.040)	0.989

All 401 participants were included in the generalized estimating equation^a^No training, in training, ISCED 4 bis 5, ISCED 6 bis 8. (*p* < 0.05) are in bold

## Discussion

We examined educational pathways taken by childhood cancer survivors and their siblings, who had previously entered parenthood. Overall, survivors and siblings who had participated in our study showed comparable attainment of educational degrees. While no significant differences were found among participating cancer survivors regarding specific diagnosis and attainment of a university degree, other factors could be correlated to academic/vocational training in survivors and siblings, such as country of origin and male gender.

Regarding the type of cancer survived by a participant, no significant differences were found in the attainment of a university degree. Improved educational outcomes in a proportion of cancer survivors could be due to increased parental support and increased motivation in terms of post-traumatic growth. The association of post-traumatic growth with educational attainment is supported by the study of Zynda et al. in which participating survivors were more likely to achieve A-levels than peers from the general population [39]. Similarly, Otth et al. found high self-management skills among CCS respondents [22].

The support system for childhood cancer patients, which includes individual support from the psychosocial team and in-hospital educators, could also play an important role in promoting patient educational skills. However, it must be noted that relatively few brain tumor patients participated in our study. In line with data from previous studies, it could have been expected that a brain tumor diagnosis and its associated treatment (e.g., surgery, irradiation) would contribute to poorer educational outcomes [21, 35]. Additionally, our participants were treated several decades ago. Current treatment strategies focus on the reduction of treatment-related toxicities that would enable cancer survivors to maintain a higher quality of life, including stricter indications for cranial irradiation. Especially in the first years of life, when the brain is particularly sensitive, cranial treatment is usually avoided. Children and adolescents who have survived cancer and treatment, especially brain tumors, require close surveillance. In the event of academic and social difficulties, these children should be offered educational rehabilitation and social skill training to maximize academic and social success.

Survivors stated more frequently than their siblings that they were currently in academic or vocational training. One reason for this could be the younger age of the cancer survivors in the present study. Another reason could be that the survivors “lost time” due to illness and treatment. Older participants would have had more time to complete their vocational training and thus, more likely to have completed vocational training at the time of survey. Siblings were less often not employed (retired, in training, etc.) or unemployed and were employed full-time more often than survivors. Overall, however, the unemployment rate of all groups was in the low single-digit percentage range and thus below the European average of 10.2% between 2010 and 2019 [37].

### Strengths and weaknesses

The strengths of this study include the large number of cancer survivors and the quality of the statistical procedures. All patients were treated according to the same GPOH treatment protocols within five participating countries. The group that directly compares patients with their biological siblings (ii) is characterized by good comparability, as some important confounding factors, such as parenting and intra-family value systems, are excluded. Thus, this group forms a good basis for generalized estimating equations. The ISCED grouping as used in our study ensures comparability of participant education in varying countries. We did, however, not include the full ISCED level (school education (ISCED 1–3) was not surveyed) and only focused on vocational education (ISCED 4–8).

There are several limitations to this study. Participants were partly recruited from previous studies that examined survivor and sibling offspring—potentially causing selection bias. The missing non-responder analysis of the sibling cohort is another weakness. In addition, except in Switzerland, the siblings were recruited by the survivors, which could also result in a possible selection bias. The smaller number of siblings compared to cancer survivors significantly reduces the group studied for the generalized estimating equation. The distribution of country of origin and cancer diagnosis differs from that of the general population. For example, participants from Germany are overrepresented and participants with brain tumor are underrepresented.

### Outlook

For many people, proper academic/vocational training and a fulfilled desire to have children are central components of a good quality of life. Young women and men usually strive to complete their education and secure their career before starting a family. It could be hypothesized that experiencing severe illness may have rendered childhood cancer survivors more mature than other young adults and may approach opportunities regarding educational pathways and starting a family with a different seriousness and appreciation. Although survivors may have lost time due to illness and treatment, and often had to deal with late effects such as fertility impairment, we did not observe differences in educational pathways that correlated with specific diagnoses. However, special attention is required for subgroups such as brain tumor patients or those who received cranial irradiation. As there are no differences in vocational education data between survivors and siblings with biological children in the present study, affected persons can be reassured that family planning and vocational education are well compatible. Since cancer treatment has improved considerably in recent decades regarding the reduction of late effects and will presumably continue to improve, cancer patients can be encouraged by the possibilities of successful, post-therapeutic academic/vocational training.

## Data Availability

The datasets generated during and/or analysed during the current study are available from the corresponding author on reasonable request.
